# Data First: Family courts data - An exploratory analysis of the nature and extent of repeat use of the family courts from 2011 to 2020 in England and Wales

**DOI:** 10.23889/ijpds.v8i2.2319

**Published:** 2023-09-14

**Authors:** Georgina Eaton, Eke Bont

**Affiliations:** 1 Ministry of Justice, London, United Kingdom

## Objectives

As people move through the courts and other justice services a wealth of administrative data is created which can provide critical new insights on justice system users, their pathways, and outcomes. Data linkage and widening access can maximise its value for research in the public good and to inform policy.

## Methods

Data linkage has, for the first time, matched parties involved in family and civil law to criminal justice, enabling cross-cutting research opportunities. This data is available to researchers via Trusted Research Environments and these partnerships can build our capacity to derive policy-relevant findings. The administrative data from the family courts in England and Wales provides a joined-up picture of people involved in family law cases such as public law, private law, adoption, Family Law Act, and divorce. The team have published research showcasing the potential of this data and the presentation will primarily focus on this work.

## Results

The family court dataset has enabled, for the first time, the extent and nature of repeat users to be explored at scale for research. This analysis provides better understanding of the stability of outcomes for children where courts make decisions about their care. We have conducted exploratory analysis of which parties in family law cases in 2011 returned over the following decade. The research investigates the frequency of return to court following involvement in different case types and roles, and transitions between case types. Locality-based analysis highlights important insights into varied patterns across England and Wales, which highlights an over-representation of family court users in some case types and roles residing in the most deprived areas in England and Wales compared to the general population.

## Conclusion

Linked administrative data can drive new insights into justice system use. Initial exploration has delivered new evidence on family justice that advances our understanding of real-world patterns, but also raises more questions. Collaboration across sectors can ensure this rich resource informs the evidence base for government policy and practice.

